# Research hotspot and trend analysis in the diagnosis of inflammatory bowel disease: A machine learning bibliometric analysis from 2012 to 2021

**DOI:** 10.3389/fimmu.2022.972079

**Published:** 2022-09-14

**Authors:** Chuan Liu, Rong Yu, Jixiang Zhang, Shuchun Wei, Fumin Xue, Yingyun Guo, Pengzhan He, Lining Shang, Weiguo Dong

**Affiliations:** ^1^Department of Gastroenterology, Renmin Hospital of Wuhan University, Wuhan, China; ^2^Department of Gastroenterology, Children’s Hospital Affiliated to Zhengzhou University, Zhengzhou, China; ^3^Department of General Surgery, The 940th Hospital of Joint Logistic Support Force of Chinese People’s Liberation Army, Lanzhou, China

**Keywords:** inflammatory bowel disease, diagnosis, precision medicine, bibliometric, keywords, CiteSpace

## Abstract

**Aims:**

This study aimed to conduct a bibliometric analysis of the relevant literature on the diagnosis of inflammatory bowel disease (IBD), and show its current status, hot spots, and development trends.

**Methods:**

The literature on IBD diagnosis was acquired from the Science Citation Index Expanded of the Web of Science Core Collection. Co-occurrence and cooperation relationship analysis of authors, institutions, countries, journals, references, and keywords in the literature were carried out through CiteSpace software and the Online Analysis platform of Literature Metrology. At the same time, the relevant knowledge maps were drawn, and the keywords cluster analysis and emergence analysis were performed.

**Results:**

14,742 related articles were included, showing that the number of articles in this field has increased in recent years. The results showed that PEYRIN-BIROULET L from the University Hospital of Nancy-Brabois was the author with the most cumulative number of articles. The institution with the most articles was Mayo Clin, and the United States was far ahead in the article output and had a dominant role. Keywords analysis showed that there was a total of 818 keywords, which were mainly focused on the research of related diseases caused or coexisted by IBD, such as colorectal cancer and autoimmune diseases, and the diagnosis and treatment methods of IBD. Emerging analysis showed that future research hotspots and trends might be the treatment of IBD and precision medicine.

**Conclusion:**

This research was the first bibliometric analysis of publications in the field of IBD diagnosis using visualization software and data information mining, and obtained the current status, hotspots, and development of this field. The future research hotspot might be the precision medicine of IBD, and the mechanism needed to be explored in depth to provide a theoretical basis for its clinical application.

## 1 Introduction

Inflammatory bowel disease (IBD) is a gastrointestinal illness that is systemic, autoimmune, recurrent, and remission, with a peak onset between the ages of 20 and 30 years (1, 2). With a frequency of more than 0.3 percent in Western populations, IBD has become a global problem (3). Ulcerative colitis (UC) and Crohn’s disease (CD) are the two main forms of IBD (4, 5), which can be distinguished by different pathological features (6). IBD patients usually have bowel symptoms, but symptoms and signs of systemic inflammation may also occur, including extraintestinal manifestations (7). Sometimes these signs and symptoms are so mild that IBD is not clinically suspected, but they may be discovered accidentally during colonoscopy for other reasons (8). IBD is currently incurable, and its treatment focuses on inducing and maintaining remission, reducing hospitalization and surgery (9).

Therefore, it is significant to diagnose IBD in a timely and correct manner. Many consensuses and guidelines have put forward clearer diagnostic criteria for IBD, but also clearly stated that the diagnosis of IBD requires careful differential diagnosis (10, 11). In recent years, with the in-depth research on the pathogenesis of IBD and the advancement of auxiliary examination techniques, there have been many new understandings of the diagnosis and differential diagnosis of IBD, which allows us to have a more comprehensive understanding of IBD and make a more accurate diagnosis. So, analyzing the clinical application progress, research status, hotspots, and development trends of IBD diagnostic research has important reference value for the extended application of IBD diagnosis in clinical practice and deeper basic research. In addition, with the deepening of research related to IBD diagnosis, the amount of literature information has grown rapidly. As a result, manual retrieval of documents has been difficult to show the full picture and progress of the research field.

Bibliometrics was defined by Pritchard in 1969 as a mathematics and statistical method to analyze literature, books and other communities. This technique, as a useful and effective tool for literature analysis, has been widely applied in different scientific knowledge domains (12). So bibliometrics is a cross-discipline that uses mathematical and statistical methods to quantitatively analyze knowledge carriers. The scientific knowledge map is a visual presentation of scientific knowledge, which can explore, draw, analyze, summarize and reveal knowledge structure and fields from the time and space dimensions (13). In recent years, the combination of knowledge visualization and bibliometrics can reveal information such as the research model, structural relationship and development process of knowledge intuitively and vividly (14, 15). Although there are many kinds of visualization analysis software such as Sci2, CiteSpace, VOS viewer, etc., CiteSpace is currently one of the most popular knowledge graph drawing tools, which is an information visualization tool developed by Professor Chen CM using Java language (16). CiteSpace has the characteristics of supporting multiple data formats, comprehensive functions and good visualization effects, and has been applied to related research in many disciplines (17).

Bibliometric has been widely used in various areas of medicine as a burgeoning method, such as internal medicine, surgery and other clinical fields (18–20). And the number of relevant bibliometrics has increased exponentially in recent years. In addition, there have been a large number of bibliometric studies on IBD. Connelly TM et al. conducted a metrological visualization analysis on 100 classic papers on UC (21). Schöffel N et al. summarized the global research output and network of UC through scientometric approach (22). Azer SA et al. conducted a bibliometric analysis of the 50 most cited IBD articles (23). However, there is no visual quantitative analysis for IBD diagnostic research. The literature in recent ten years is more referential for grasping the current research situation and guiding the research on the future trend. Therefore, based on the bibliometric method and scientific knowledge map, this study used CiteSpace software to visually analyze the international research progress and current status of IBD diagnosis in the past ten years from multiple perspectives such as document distribution and co-occurrence maps. At the same time, it revealed the research hotspots and future trends of relevant publications through the timeline view and the track of mutation words, and made some prospects for the research frontiers. In addition, we screened the scientific literature on precise diagnosis and management of IBD to provide reference and data support for international IBD diagnosis research.

## 2 Materials and methods

### 2.1 Data source

This study was a bibliometric study that did not involve any clinical trials and patient consent. Therefore, there was no need for the approval of the ethics committee or institutional review board. Data were acquired from the Science Citation Index Expanded (SCI-E) of the Web of Science Core Collection (WoSCC) of Clarivate Analytics (https://clarivate.com/) on April 28, 2022. The search time range was from January 1, 2012, to December 31, 2021, and the search was limited to publications written in the English language. In the WoSCC database, the subject term search method was used, and the subject area was not limited. The search strategy was (“inflammatory bowel disease” or “Crohn” or “Crohns-disease” or “ulcerative colitis” or “IBD” or “CD” or “UC”) and “diagnosis”. All electronic searches were performed on April 28, 2022. Two independent reviewers collected all the data by reading the titles and abstracts acquired from SCI-E of the WoSCC database. When necessary, the full texts were downloaded from PubMed or other databases. Any differences between the two reviewers were settled through discussion with a third reviewer.

### 2.2 Data establishment and processing

#### 2.2.1 Data creation and conversion

Note Express, a full-featured document management software program, was used to manage the studies obtained from different databases, with document information retrieval, download, and classification functions. The retrieved journal articles researching the diagnosis of IBD were imported into Note Express software and duplicated literature was electronically removed, further screening, and finally, 14,742 articles were included. Document data exported from WoSCC were saved in “RefWorks” format. Two researchers reviewed the selected articles, and the following data were identified and recorded for analysis: (1) titles, (2) authors, (3) citation number, (4) keywords, (5) publication year, (6) topics (7) reference and (8) institutions and countries of origin. Furthermore, the journal names and impact factors (IFs) were also recorded using the 2021 edition of the Journal Citation Reports (JCR). Data were converted to “txt” format, named download_*.txt, and then imported into CiteSpace 5.8.R3 version and the Online Analysis platform of Literature Metrology (OALM) (http://bibliometric.com/) for analysis. OALM provides researchers with bibliometric analysis of scientific citation data in the form of graphic visualization through web services, and provides valuable reference information for researchers to carry out research with the simplest operation method and the most intuitive expression method.

#### 2.2.2 Data processing

Excel software was used to analyze the included literature data. We selected appropriate parameters in the CiteSpace 5.8.R3 version to generate co-occurrence visualization maps of journals, authors, institutions, countries, references, and keywords for IBD diagnostic research, and performed cluster analysis and emergence analysis of keywords. ①Time slicing: 2012-2021; time zone selection (year per slice): 1 year; node type: author, institution, country, reference, cited author, cited journal, keyword. ②The threshold (top N per slice): 10%, that is, the top 10% but less than 100 high-frequency keywords were selected in each time zone. In order to prevent the co-citation network from being too complex, a Pathfinder algorithm was used in this paper, which could simplify the network by removing the edges that violate the triangle inequality and accurately extract the key structure of the network. The default system was selected for visualization. CiteSpace standardized algorithms included the Cosine similarity algorithm, Jaccard similarity algorithm, and Dice similarity coefficient. The next step was to analyze the relationship between the data. The software used the method of cluster analysis to reveal the correlation. CiteSpace clustering algorithm mainly used nominal terms to detect research hotspots, which could help researchers find mutation words in the map, explore research hotspots, and grasp the research direction. There were three Clustering algorithms: Clustering algorithm, LLR algorithm, and MI algorithm. At the same time, OALM was used to analyze the number of common national articles by year, the number of common keywords by year, partnerships (including authors, institutions, and countries) and article citation relationships.

## 3 Results

### 3.1 The number of literature and general characteristics

**Figure 1:**
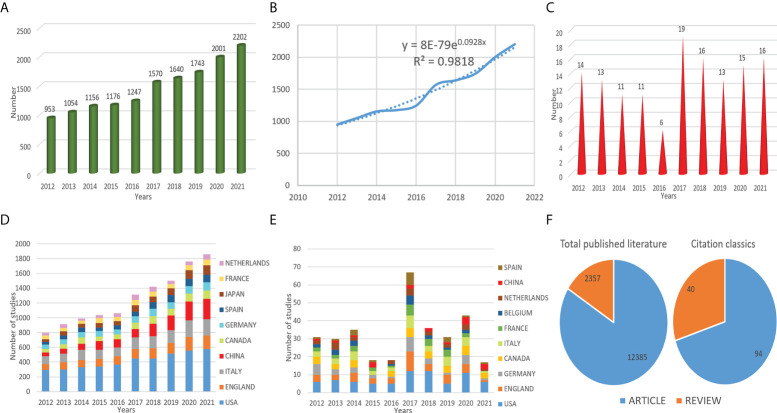
Annual number of publications **(A)**, exponential growth graph of number of publications **(B)**, the number of publications per year in common countries **(D)** of IBD diagnostic studies in the past ten years, and annual number of publications **(C)**, the number of publications per year in common countries **(E)** of citation classics, and the type of literature **(F)**.

Based on WoSCC, it was found that the overall trend of the number of published articles was increasing from 2012 to 2021, indicating that the research on the diagnosis of IBD had received attention and the value of mining was getting higher and higher (**Figure 1A**). At present, the number of articles published in 2021 had reached the maximum, with a total of 2,202 articles. According to Price’s law, the growth of literature production was exponential, and the exponential curve equation was y = 8E-79e^0.0928x^. The simulation curve fits well with the annual literature growth trend with a high coefficient of determination (R^2^ = 0.9818) (**Figure 1B**). By the fitting curve, we can predict that the annual articles will continue to grow in the coming years. According to the exponential curve equation, the average annual growth rate was 9.28%. According to the calculation of the mathematical formula, the literature doubling time was 7.47 years. In addition, we selected 134 highly cited and hot papers in this field in the past ten years, and referred to these as “citation classics” (**Supplementary Table 1**). Among them, the number of articles published in 2017 was the most, reaching 19 (**Figure 1C**). Most of the document types were articles (**Figure 1F**). In addition, based on OALM, the number of published articles in common countries had been counted year by year (**Figures 1D, E**).

### 3.2 Author analysis

**Figure 2:**
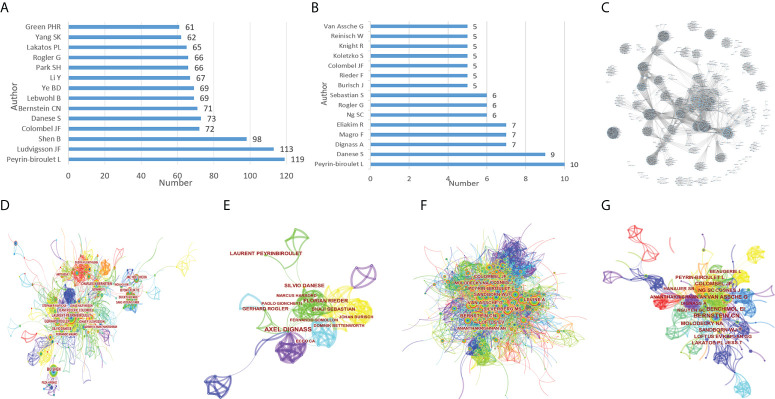
The number of articles published by common authors **(A)**, the co-occurrence of authors **(D)**, the co-occurrence of cited authors **(F)** of IBD diagnostic studies in the past ten years, and the number of articles published by common authors **(B)**, the co-occurrence of authors **(E)**, the co-occurrence of cited authors **(G)** and the cooperation relationship between authors **(C)** of citation classics.

Based on WoSCC, there were 62,916 authors involved in relevant research in the past ten years, and 1,605 authors were involved in citation classics (**Figures 2A, B**). The cooperative network of the authors and the cited authors was analyzed through CiteSpace, and the co-occurrence visualization maps were obtained. Based on CiteSpace, the analysis was as follows: There were 702 nodes in the research in the past ten years, representing 702 authors (**Figure 2D**), and there were 280 nodes in citation classics (**Figure 2E**). The larger the node was, the more articles the author published. Among them, the author with the highest cumulative number of articles was Peyrin-biroulet L, with 119 articles. Based on the Web of Science, the high citations index (h-index) is a mixed quantitative index, which can be used to evaluate the number and level of academic output of researchers. Also based on the Web of Science, the median citation percentile can reflect the influence of the author. The author of Colombel JF was the most influential, with an h-index of 125 and a median citation percentile of 87th (**Table 1**). In the past ten years of research, there were 1,034 cited authors (**Figure 2F**), and 382 of the citation classics (**Figure 2G**). There was a cooperative relationship between highly productive authors and a stable research team had been formed. At the same time, the author’s cooperation network diagram also showed that half of the research teams have little cooperation (**Figure 2C**).

**Table 1:** Top 14 authors of relevant literature of IBD diagnosis in nearly 10 years.

Rank	Authors	Frequency	H-index	Median citation percentile	Centrality	Degree
1	Peyrin-biroulet L	119	88	72nd	0.09	46
2	Ludvigsson JF	113	64	76th	0.04	25
3	Shen B	98	52	53rd	0.05	23
4	Danese S	73	93	77th	0.08	44
5	Colombel JF	72	125	87th	0.07	41
6	Bernstein CN	71	88	76th	0.08	35
7	Lebwohl B	69	37	62nd	0.02	14
8	Ye BD	69	36	57th	0.01	22
9	Li Y	67	5	41st	0.00	3
10	Park SH	66	29	62nd	0.02	19
11	Rogler G	66	67	62nd	0.09	40
12	Lakatos PL	65	17	53rd	0.08	49
13	Yang SK	62	46	57th	0.02	23
14	Green PHR	61	73	73rd	0.00	11

### 3.3 Country and institution analysis

**Figure 3:**
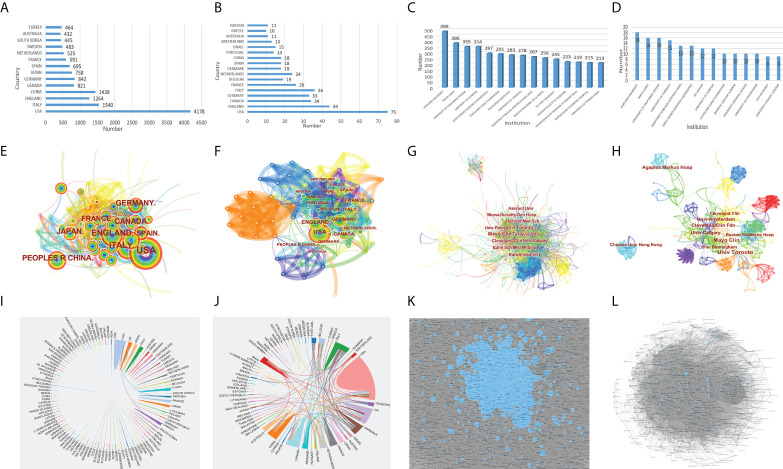
The number of articles published by common countries **(A)**, the co-occurrence of countries **(E)**, the cooperation relationship between countries **(I)** of IBD diagnostic studies in the past ten years, and the number of articles published by common countries **(B)**, the co-occurrence of countries **(F)**, the cooperation relationship between countries **(J)** of citation classics; the number of articles published by common institutions **(C)**, the co-occurrence of institutions **(G)**, the cooperation relationship between institutions **(K)** of IBD diagnostic studies in the past ten years, and the number of articles published by common institutions **(D)**, the co-occurrence of institutions **(H)**, the cooperation relationship between institutions **(L)** of citation classics.

Based on WoSCC, there were 149 countries/regions and 11,324 institutions in the relevant research in the past ten years. Among them, the USA published the most articles, reaching 4178 articles (**Figure 3A**), and Harvard University had the most articles, reaching 488 articles (**Figure 3C**). At the same time, 65 countries and 826 institutions were involved in the citation classics. The USA published the most articles with 75 articles (**Figure 3B**), and Harvard University had the most articles with 18 articles (**Figure 3D**). The cooperative networks of the countries and the institutions were analyzed through CiteSpace, and the co-occurrence visualization maps were obtained. Based on CiteSpace, the analysis was as follows: In the past ten years, there were 180 countries/regions (**Figure 3E**). Among them, the USA had the most published articles with 4,020 articles, followed by ITALY, CHINA, ENGLAND, GERMANY, and CANADA, all with more than 500 articles. Among the citation classics, there were 65 countries, with the USA leading the list with 75 papers, and 7 countries had published more than 20 papers (**Figure 3F**). There were 563 institutions in the literature in the past ten years (**Figure 3G**). Among them, Mayo Clin had the most published articles with 358 articles, followed by Karolinska Inst, Univ Toronto, Tel Aviv Univ, Harvard Med Sch, Cleveland Clin, Massachusetts Gen Hosp, Univ Milan, Icahn Sch Med Mt Sinai, Univ Penn, and Univ Calgary, all with more than 100 articles. Among the citation classics, there were 263 institutions, of which Mayo Clin had published the most with 13 articles, and there were 9 institutions with articles number ≥ 5 (**Figure 3H**). Based on OALM, each small black dot represented an institution, and the connection represented mutual cooperation. It could be seen that the institutions were closely connected, and most of the research institutions were universities (**Figures 3K, L**). Countries had stable cooperative relations, but most countries had fewer connections (**Figures 3I, J**).

### 3.4 Citation analysis of articles and journals

**Figure 4:**
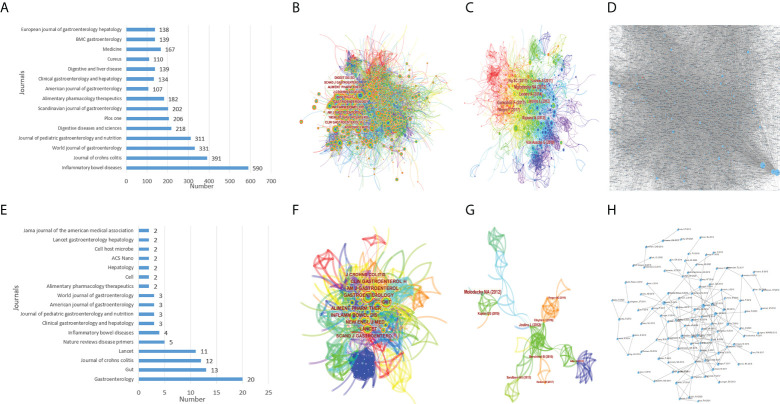
The number of aricles publishe by journals **(A)**, the co-occurrence of journals **(B)**, the co-occurrence of cited journals **(C)**, the co-occurrence relationship between journals **(D)** of IBD diagnostic studies in the past ten years, and the number of articles published by journals **(E)**, the co-occurrence of journals **(F)**, the co-occurrence of cited journals **(G)**, the cooperation relationship between journals **(H)** of citation classics.

Based on WoSCC, related researches in the past ten years had been published in 2,771 journals. Inflammatory bowel diseases (2021 IF=7.29) contributed the most to this research field, reaching 590 articles (**Figure 4A**). Among the citation classics, 59 journals were involved, and Gastroenterology (2021 IF= 33.88) published the most articles, reaching 20 articles (**Figure 4E**). The cooperative networks of the cited journals and references were analyzed through CiteSpace, and the co-occurrence visualization maps were obtained. Based on CiteSpace, the analysis was as follows: In the past ten years, 1,462 journals were cited, of which Gastroenterology was cited the most, up to 6576 times, and 17 journals were cited more than 2000 times (**Figure 4B**). There were 313 journals cited in citation classics, of which Gastroenterology was the most cited, reaching 100 times, and there were 14 journals with ≥ 50 citations (**Figure 4F**). In the recent ten years of research, the article (24) had been cited the most, up to 268 times, and ≥100 times were 18 studies (**Figure 4C**). Among the citation classics, the article (24) also was cited the most, reaching 12 times, and ≥5 times were 8 studies (**Figure 4G**). In the article citation relationship network diagram based on OALM, each small black dot represented a study, and the lines represented mutual citations. It could be seen that the mutual references were relatively close, especially in the studies in the past ten years (**Figures 4D, H**).

### 3.5 Keywords analysis

#### 3.5.1 Word frequency analysis

Keywords play the role of condensing and generalizing the theme of the literature. Counting the keywords in the research literature and obtaining keywords with more citations, reflects the importance of the keywords to a certain extent, and can be used to analyze the evolution of research hotspots (25). Through the analysis of keywords, in addition to the search terms “inflammatory bowel disease”, “Crohn’s disease”, “ulcerative colitis” and “diagnosis”, this study also presented keywords that reflected IBD-related diseases, such as “colorectal cancer”, “clostridium difficile infection”, “primary sclerosing cholangitis” etc.; reflected IBD treatment, such as “management” and “therapy”; reflected IBD related to features, such as “quality of life” “risk factor” “natural history” “evidence based consensus” “follow up” “children” etc. (**Table 2**).

**Table 2:** Top 12 keywords of relevant literature of IBD diagnosis.

Rank	Related researches in nearly 10 years				Rank	Citation classics			
	Keywords	Freq	Keywords	Centrality		Keywords	Freq	Keywords	Centrality
1	inflammatory bowel disease	4281	Inflammatory bowel disease	0.03	1	inflammatory bowel disease	56	inflammatory bowel disease	0.78
2	Crohn’s disease	3250	diagnostic accuracy	0.03	2	ulcerative colitis	41	Crohn’s disease	0.73
3	ulcerative colitis	2601	natural history	0.02	3	Crohn’s disease	34	ulcerative colitis	0.2
4	diagnosis	2042	rheumatoid arthritis	0.02	4	quality of life	10	colorectal cancer	0.16
5	management	1098	clostridium difficile infection	0.02	5	colorectal cancer	9	rheumatoid arthritis	0.12
6	prevalence	739	antibody	0.02	6	clostridium difficile infection	8	quality of life	0.09
7	risk	649	prediction	0.02	7	risk factor	7	risk factor	0.09
8	children	627	calprotectin	0.02	8	management	7	primary sclerosing cholangitis	0.07
9	risk factor	556	ct enterography	0.02	9	evidence based consensus	6	clostridium difficile infection	0.06
10	therapy	554	receptor	0.02	10	primary sclerosing cholangitis	6	chronic kidney disease	0.06
11	epidemiology	454	prognosis	0.02	11	diagnosis	5	genome wide association	0.06
12	colorectal cancer	429	genome wide association	0.02	12	follow up	5	diagnosis	0.05

**Figure 5:**
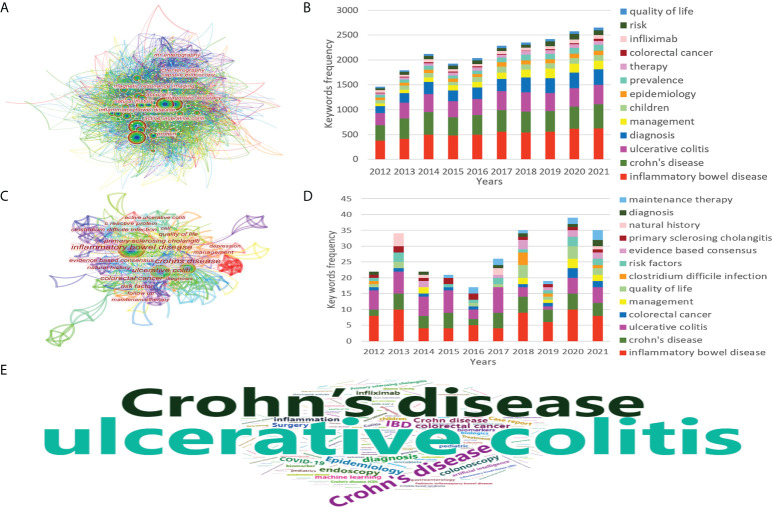
The co-occurrence of keywords **(A)**, annual number of common keywords **(B)** of IBD diagnostic studies in the past ten years, and the co-occurrence of keywords **(C)**, annual number of common keywords **(D)** of citation classics, and keywords cloud form in PubMed **(E)**.

Visual graph analysis was carried out by CiteSpace software, which contained 818 nodes, one of which represented a keyword. The larger the node, the higher the frequency of the keyword. The connection between nodes represented the connection between keywords, and the thicker the connection, the higher the frequency of keyword co-occurrence and the closer the connection. The color of the link represented the time when the keyword appeared, with cool colors appearing earlier and warm colors appearing later. Node centrality was to measure the importance of a node in the network and the closeness of its connection with other nodes. The higher the centrality of the keyword, the easier it was to become a key node in the network, indicating that this keyword had influence in this field and the scope of research surrounding this keyword was wide. There were 818 keywords obtained in the research in the past ten years, of which there were 5 keywords with word frequency ≥1000 (**Figure 5A**), and there were 266 keywords obtained in citation classics, 4 of which have a word frequency ≥10 (**Figure 5C**). The influential keywords in the visualization map included inflammatory bowel disease (0.78), Crohn’s disease (0.73), ulcerative colitis (0.2), colorectal cancer (0.16), rheumatoid arthritis (0.12), etc. (**Table 2**). This study also visualized the evolution of common keywords year by year (**Figures 5B, D**). In addition, in order to strengthen the reliability of key keywords, we searched 1,000 researches related to this field in PubMed, which contained 2,127 keywords. After removing some redundancy, there were 1907 keywords. The number of keywords appearing in these articles was displayed in the form of a word cloud (**Figure 5E**), and it could be seen that the trend of core keywords was roughly the same as what we had mentioned above.

#### 3.5.2 Cluster analysis

**Figure 6:**
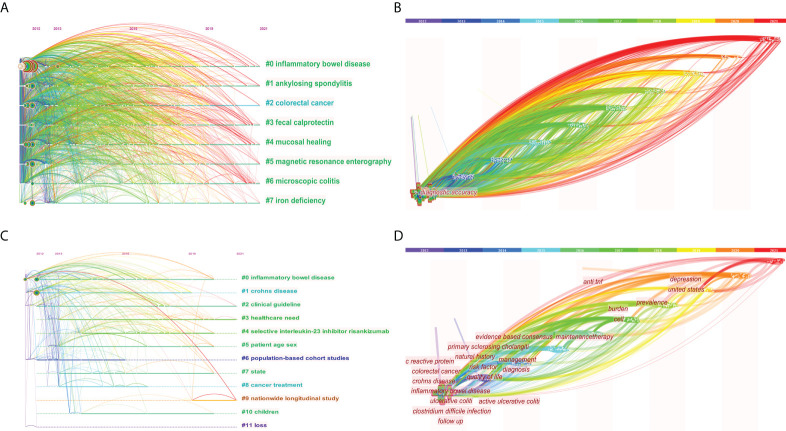
The clustering timeline view **(A)** and time zone map **(B)** of keywords on IBD diagnostic studies in the past ten years, and the clustering timeline view **(C)** and time zone map **(D)** of keywords of citation classics.

Co-word clustering was to perform correlation operations on keywords that co-occur in research articles, and cluster closely related words into categories, so as to achieve the purpose of mining hidden information. In this paper, a cluster analysis of the main keywords was performed through CiteSpace and the log-likelihood ratio (LLR) method was used to label the clusters, so as to obtain the clustering timeline view (**Figures 6A, C**) and time zone map (**Figures 6B, D**). There were two important evaluation indicators for the effect of cluster map drawing, namely Modularity Q (Q, value interval [0,1]) and Weighted Mean Silhouette S (S, value interval [-1,1]). The Q value was used to evaluate the goodness of the clustering network, and the higher the value, the better the structure of the clustering network. Q>0.3 indicated that the obtained network structure was significantly convincing. The S value was used to measure the homogeneity of cluster members, and the larger the value, the higher the consistency between members within the class. S>0.5 meant that the clustering result was reasonable. The Q values in **Figure 6** were 0.3876 and 0.591 (both> 0.3), and the S values were 0.7108 and 0.8683 (both> 0.7), indicating that these clustering diagrams were reasonable and had reference value.

In the past ten years of research, 8 meaningful clusters were formed (**Table 3A**), and 12 clusters were formed in citation classics (**Table 3B**). From #0 to #12, the smaller the number, the more keywords were included in the cluster. There were multiple overlapping clusters in the keyword clustering graph, indicating that they were closely related. The clustering timeline view visually displayed the time span of each cluster and the correlation between different clusters, so as to clearly showed the evolution process of the research. The abscissa (x-axis) of the timeline view was the year of publication, and the ordinate (y-axis) was the cluster number. The keywords time zone map could clearly show the evolution process of knowledge in the time dimension, obtain the development trend of research hotspots, and provide directions for future research. The cluster analysis in this paper found that the research mainly focused on two aspects: ①research on related diseases caused or coexisting with IBD, such as colorectal cancer and autoimmune diseases; ②research on diagnosis and treatment of IBD, such as imaging diagnosis and maintenance therapy.

**Table 3:** Keywords co-occurrence network clustering table.

Category	Cluster ID	Size	Silhouette	Mean (Year)	Top terms (top 5)
A. Related researches in nearly 10 years	0#	140	0.694	2014	inflammatory bowel disease; natural history; population-based study; clinical characteristics; disease course
	1#	127	0.654	2014	ankylosing spondylitis; inflammatory diseases; axial spondyloarthritis; rheumatoid arthritis; pyoderma gangrenosum
	2#	126	0.673	2013	colorectal cancer; colorectal neoplasia; Crohn’s colitis; Crohn’s disease; ulcerative colitis-associated neoplasia
	3#	117	0.704	2015	fecal calprotectin; faecal calprotectin; fecal lactoferrin; volatile organic compound; intestinal tuberculosis
	4#	99	0.678	2014	mucosal healing; exclusive enteral nutrition; long-term efficacy; clinical remission; serious infection
	5#	96	0.807	2013	magnetic resonance enterography; capsule endoscopy; MR enterography; small bowel; inflammatory bowel disease
	6#	80	0.756	2015	microscopic colitis; collagenous colitis; lymphocytic colitis; inflammatory bowel disease; Crohn’s disease
	7#	35	0.864	2015	iron deficiency; iron deficiency anemia; ferric carboxymaltose; Crohn’s disease; ulcerative colitis
B. Citation classics	0#	43	0.797	2015	inflammatory bowel disease; toronto consensus statement; worldwide incidence; environmental risk factor; 21st century
	1#	30	0.932	2014	Crohn’s disease; risk pathogenesis prevention; reduced risk; IBD detection; initial diagnosis monitoring
	2#	29	0.864	2015	clinical guideline; fecal microbiota transplantation; therapeutic potential; review article; gut microbiome
	3#	29	0.8	2017	healthcare need; unmet need; hidradenitis suppurativa; evaluating patient; global survey
	4#	25	0.831	2017	selective interleukin-23 inhibitor Risankizumab; to-severe Crohn’s disease; double-blind placebo-controlled phase; induction therapy; international expert
	5#	21	0.901	2016	patient age sex; phenotype associate; young adult; increasing incidence; rectal cancer
	6#	20	0.876	2012	population-based cohort studies; colorectal cancer; decreasing risk; declining risk; colitis epidemiology study
	7#	19	0.876	2015	state; burden; functional gastrointestinal disorder; childhood; gastrointestinal liver
	8#	19	0.903	2015	cancer treatment; role; cancer; iron deficiency anemia; Crohn’s disease
	9#	12	0.97	2020	nationwide longitudinal study; dementia risk; inflammatory bowel disease; Crohn’s disease; systematic review
	10#	9	0.945	2015	children; ESPGHAN; Porto criteria; adolescent; diagnosis
	11#	6	0.973	2012	loss; infantile inflammatory bowel disease; therapy; diagnosis; inflammatory bowel disease
C. Studies of Precision Diagnosis and Management	0#	40	0.773	2016	IBD research; pragmatic clinical research; mucosal healing; deep remission; endoscopic diagnosis
	1#	34	0.765	2016	inflammatory diseases; omics approaches; focal adhesion complex; fecal calprotectin level; serological responses
	2#	34	0.867	2004	multicenter national study; saudi arabia; pediatric inflammatory bowel disease; mucosal damage; quantitative assessment
	3#	33	0.964	2008	new serological biomarker; Crohn’s disease; clinical aspect; inflammatory bowel disease; community-based survey
	4#	33	0.936	2019	oral delivery; IBD-what why; precision medicine; mandatory next step; network medicine
	5#	32	0.86	2014	biological marker; faecal calprotectin; colorectal malignancy; diagnostic precision; mood disorder
	6#	29	0.902	2012	observational study; abdominal discomfort; genotype influence disease phenotype; inflammatory bowel; fecal calprotectin
	7#	29	0.881	2016	learning health system; early biologic therapy; biomarker-based model; precision medicine; Crohn’s disease
	8#	21	0.976	2014	clinical laboratory application; neutralizing antibody response; reporter-gene assay; functional activity; evaluating appropriateness
	9#	15	0.923	2015	Bayesian regression approach; prioritization; complex diseases; candidate domain; salivary microbiome
	10#	10	0.988	2011	validation; quantification; mycobacterium avium subsp paratuberculosis; specific detection; standardization
	11#	9	1	2010	therapy evaluation; radiolabeled anti-tnf alpha; role; diagnostic purposes; monoclonal antibodies
	12#	8	0.967	2012	assessment; wireless capsule; Crohn’s disease lesion; inflammatory bowel disease; precision medicine

#### 3.5.3 Emergent analysis

Kleinberg’s burst detection algorithm can be used to detect the sudden growth of research interest in a discipline (26). Burst detection is an original computational technique that can detect drastic changes in events. A burst of an event means the event appearance shows a surge increase, such as in the frequency of a keyword. And burst keywords may be a key direction in development trends. So, keywords emergence analysis could show that the research of a keyword had increased during a certain period of time, which meant that the attention of the keyword had increased during this period of time. The analysis of emergent words in the literature related to IBD diagnosis from 2012 to 2021 was conducted through CiteSpace software. Each emergence word had a highlight bar composed of each cell, and each cell represented a year (**Table 4**). In the early period of 2012-2021, IBD diagnostic research mostly lay in general characteristics and diagnostic methods. In the later period, the research hotspot of IBD diagnosis expanded to research on the treatment and mechanism of IBD, and this aspect of research needed to be further explored. It was worth noting that precision medicine might be the research trend in the future.

**Table 4:** Emergent analysis of keywords in related literature of IBD diagnosis.

Category	Keywords	Strength	Begin	End	2012 - 2021
A. Related researches in nearly 10 years	CT enterography	10.96	2012	2013	▃▃▂▂▂▂▂▂▂▂
	survival	5.83	2012	2013	▃▃▂▂▂▂▂▂▂▂
	carcinoma	5.31	2012	2013	▃▃▂▂▂▂▂▂▂▂
	cancer risk	5.3	2012	2013	▃▃▂▂▂▂▂▂▂▂
	MR enteroclysis	5.24	2012	2013	▃▃▂▂▂▂▂▂▂▂
	increasing incidence	6.2	2013	2014	▂▃▃▂▂▂▂▂▂▂
	liver disease	5.79	2015	2016	▂▂▂▃▃▂▂▂▂▂
	tumor	5.53	2015	2018	▂▂▂▃▃▃▃▂▂▂
	regulatory T cell	4.95	2015	2016	▂▂▂▃▃▂▂▂▂▂
	diet	5.71	2017	2019	▂▂▂▂▂▃▃▃▂▂
	postoperative complication	5.3	2017	2018	▂▂▂▂▂▃▃▂▂▂
	combination therapy	4.98	2017	2021	▂▂▂▂▂▃▃▃▃▃
	pathway	6.11	2018	2021	▂▂▂▂▂▂▃▃▃▃
	fecal microbiota	5.75	2018	2019	▂▂▂▂▂▂▃▃▂▂
	anti TNF therapy	5.74	2018	2021	▂▂▂▂▂▂▃▃▃▃
	gut microbiota	5.19	2018	2021	▂▂▂▂▂▂▃▃▃▃
	burden	5.7	2019	2021	▂▂▂▂▂▂▂▃▃▃
	clinical practice guideline	5.62	2019	2021	▂▂▂▂▂▂▂▃▃▃
	Care	5.52	2019	2021	▂▂▂▂▂▂▂▃▃▃
	accuracy	5.13	2019	2021	▂▂▂▂▂▂▂▃▃▃
	evidence based consensus	4.88	2019	2021	▂▂▂▂▂▂▂▃▃▃
	precision medicine	1.68	2020	2021	▂▂▂▂▂▂▂▂▃▃
B. Citation classics	natural history	1.99	2013	2013	▂▃▂▂▂▂▂▂▂▂
	primary sclerosing cholangitis	1.9	2013	2016	▂▃▃▃▃▂▂▂▂▂
	maintenance therapy	1.52	2016	2017	▂▂▂▂▃▃▂▂▂▂
	quality of life	1.85	2018	2021	▂▂▂▂▂▂▃▃▃▃
	necrosis factor alpha	1.66	2018	2018	▂▂▂▂▂▂▃▂▂▂
	clostridium difficile infection	1.64	2018	2018	▂▂▂▂▂▂▃▂▂▂
	risk	1.59	2020	2021	▂▂▂▂▂▂▂▂▃▃
	diagnosis	1.44	2020	2021	▂▂▂▂▂▂▂▂▃▃
C^#^	intestinal inflammation	2.8	2006	2011	(1990-2021)
	maintenance therapy	3.19	2019	2021	(1990-2021)
	precision medicine	2.95	2020	2021	(1990-2021)

C^#^: Studies of Precision Diagnosis and Management;

A: γ[0,1]=2; Minimum Duration=2; B/C: γ[0,1]=0.6; Minimum Duration=1.

### 3.6 Literature analysis on precision diagnosis and management of IBD

Through the above analysis, we found that precision medicine might be the future research direction, and we have now entered the era of personalized medicine. Therefore, we also searched and included 96 articles related to precise diagnosis and management of IBD for analysis after inclusion and exclusion criteria.

#### 3.6.1 General feature distribution

**Figure 7:**
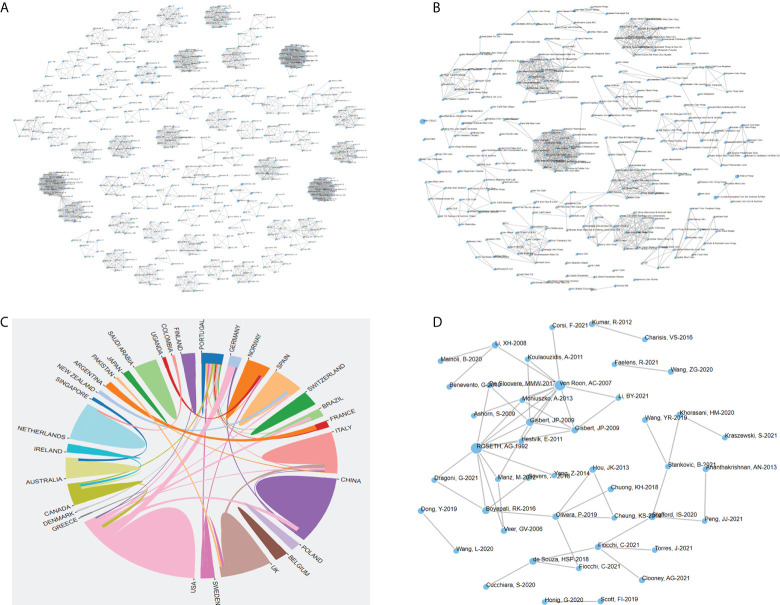
The cooperation relationship between authors **(A)**, institutions **(B)**, countries **(C)**, and articles **(D)** of the literature on precision diagnosis and management of IBD.

It was found that the related research was still mainly produced in the United States, and then China ranked second. The institution with the most articles was Katholieke Univ Leuven, with 4 articles, and the author with the most articles was CLAUDIO FIOCCHI, with 3 articles (**Supplementary Table 2**). The most published journal was Inflammatory Bowel Diseases, with 11 articles. Based on OALM to analyze the cooperation relationship, including the relationship between authors (**Figure 7A**), institutions (**Figure 7B**), and countries (**Figure 7C**), it could be seen that there were fixed cooperative relationships. At the same time, article citation relationship network analysis was also carried out (**Figure 7D**).

#### 3.6.2 Keywords analysis

**Figure 8:**
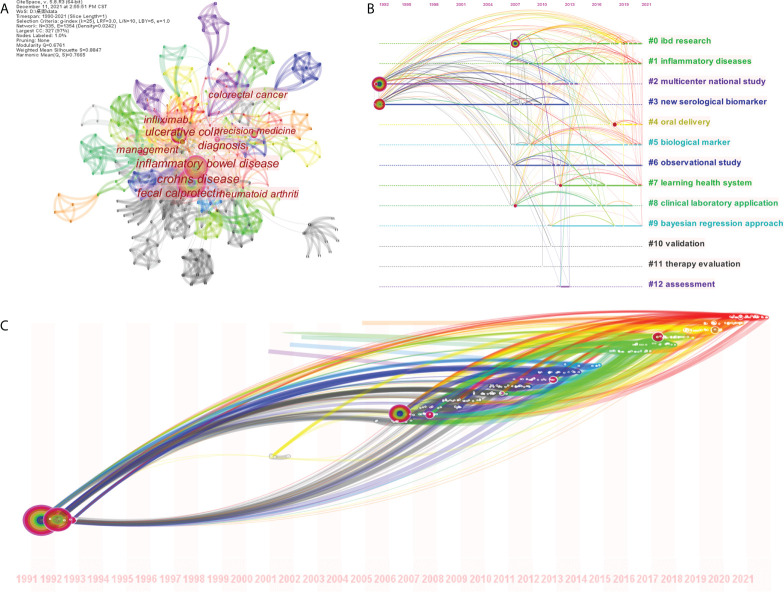
The co-occurrence of keywords **(A)**, the clustering timeline view of keywords **(B)** and time zone map of keywords **(C)** of the literature on precision diagnosis and management of IBD.

335 keywords were discovered based on CiteSpace (**Figure 8A**). Σ (sigma) was an index of the degree of dispersion of research keywords. The higher the Σ, the stronger the correlation between the keyword and the research. We analyzed the top 12 keywords sorted by word frequency, centrality, and Σ (**Table 5**). The keywords were clustered analysis, and the clustering timeline view (**Figure 8B**) and time zone map (**Figure 8C**) were obtained. 13 clusters were formed (**Table 3C**), which were mainly concentrated on the method exploration and evaluation of precision diagnosis and management of IBD, of which 2006 was an important node. The emergence analysis showed that the future hotspots were “maintenance therapy” and “precision medicine”, especially the latter (**Table 4C**).

**Table 5:** Top 12 keywords of the literature on precision diagnosis and management of IBD.

Rank	Keywords	Freq	Keywords	Centrality	Keywords	Σ
1	inflammatory bowel disease	59	Crohn’s disease	0.53	precision medicine	1.59
2	Crohn’s disease	56	inflammatory bowel disease	0.35	diagnosis	1.52
3	ulcerative colitis	32	diagnosis	0.3	intestinal inflammation	1.24
4	management	20	ulcerative colitis	0.23	infliximab	1.2
5	diagnosis	18	fecal calprotectin	0.23	system	1.13
6	precision medicine	13	management	0.2	maintenance therapy	1.1
7	fecal calprotectin	13	rheumatoid arthritis	0.2	risk	1.06
8	infliximab	11	precision medicine	0.17	gut microbiota	1.04
9	rheumatoid arthritis	9	colorectal cancer	0.14	induction	1.03
10	colorectal cancer	8	infliximab	0.12	diagnostic precision	1.02
11	c reactive protein	7	C reactive protein	0.09	pathogenesis	1.02
12	maintenance therapy	7	intestinal inflammation	0.08	Crohn’s disease	1

## 4 Discussion

According to a report by Kaplan in 2015, there were more than 1 million people in the Americas and 2.5 million people in Europe with IBD (27). The lack of epidemiologic IBD data from developing countries suggested that the situation could be even worse, given an increase in incidence associated with recent industrialization (28). Early diagnosis and treatment of IBD could usually improve the quality of life of these patients, so its diagnostic research is valuable. It is particularly urgent to grasp the overview of the research field in today’s massive literature, and bibliometrics can meet this demand.

This article was the first based on the bibliometric method combined with the content analysis method to conduct a mathematical statistical analysis of the literature related to IBD diagnosis, and to mine research hotspots and development trends based on data information. Generally speaking, in order to obtain a standard CiteSpace knowledge spectrum, four processes were required: software installation, original article retrieval and download, software functional operation (including adjustment of various parameters, etc.), and graph interpretation. To collect articles accurately and comprehensively, authors should pay attention to data retrieval methods and select appropriate parameters according to their own needs, and adjust the messy spectrum to obtain a clear and beautiful spectrum.

An analysis of 14,742 articles related to IBD diagnosis included in this study from 2012 to 2021 found that the number of publications was generally on the rise, which showed that the research had prospects and potential. The number of articles published in the top 10 countries accounted for 80% of all articles, and 90% of these 10 countries were developed countries, because these countries generally had advanced medical research institutions and top medical researchers. The government had the ability to establish a complete medical security system and provided sufficient funds for drug research. Among them, the United States was far ahead and had played a leading role, which might be related to the US financial resources for scientific research (29). Canada has been the fourth largest IBD research funder in the world, investing over $119 million from 2013 to 2017. With this investment, on all measures of academic productivity and influence, Canada ranks in the top two or three internationally (30). China was the only developing country among these 10 countries, which showed that China had made great progress in this research recently. China’s investment in research and invention had surpassed that of any other country except the United States, and its funding was growing at a rate of 20% per year (31), leading to significant progress in many medical research fields (32, 33). At present, the author with the most articles on IBD diagnostic research was PEYRIN-BIROULET L from the University Hospital of Nancy-Brabois. Surprisingly, in both the analysis of total literature and citation classics, the author not only performed well in the number of publications but also in the quality of publications, which showed that he played a pivotal role in this research. Mayo Clin, Univ Toronto and Karolinska Inst were institutions with a large number of articles, and they belonged to the United States, Canada and Sweden, respectively. In addition, the United States had played a key role in promoting international cooperation, with the strongest cooperation between the United States and Canada. So, it could be found that scientists, institutions and countries all benefit from international cooperation (34). Due to the attractiveness of high IF journals to the scientific community (35), the IF of journals was in some way one of the most powerful citation indicators. Highly cited articles were usually published in high-IF journals. Researches in this field were mainly published in Inflammatory Bowel Diseases and Journal of Crohn’s colitis, with IFs of 7.29 and 10.02 respectively, which also hinted at the quality of output in this field.

Visualized analysis of keywords in the literature related to IBD diagnosis through CiteSpace revealed that the hot words were mainly “colorectal cancer”, “rheumatoid arthritis”, “clostridium difficile infection”, “primary sclerosing cholangitis”, “risk”, “therapy”, “maintenance therapy”, “quality of life”, “antibody”, “evidence-based consensus”, “diagnostic accuracy”, etc. With the continuous deepening and expansion of research, precision medicine related to “diagnostic accuracy” had become a new hot spot and trend. In the early stage of IBD diagnostic research, the general characteristics and diagnostic methods were mostly focused on. In the later stage, the research hotspot expanded to the treatment mechanism of IBD and the quality of life. The analysis of citation classics further verified the above situation.

In classifying the clustering fields, the disease fields had the highest percentages, such as infections and tumors. These diseases were mainly focused on related diseases caused by or coexisting with IBD, such as colorectal cancer and autoimmune diseases. These diseases might be related to the pathogenesis and complications of IBD. There was evidence that the diversity of the gut microbiota in IBD decreased and mucosal adherent bacteria increased (36). Changes in the intestinal microbiota led to a serious imbalance of host physiology and immune homeostasis, which ultimately led to infection and inflammation. For example, patients with IBD were particularly vulnerable to Clostridium difficile infection (CDI), with increased morbidity and mortality (37). Clostridium difficile is an intestinal pathogen, which is considered to be the main cause of antibiotic-related diarrhea and colitis, and also an important complication of IBD (38). One of the extraintestinal manifestations of IBD is primary sclerosing cholangitis (PSC), and its main clinical manifestations are related to IBD (39). Approximately 70% of patients with PSC have potential IBD, and UC is the most common in more than 75% of cases (40). PSC and IBD are interrelated diseases, mainly immune-mediated processes (41). These two diseases have common antibodies. For example, perinuclear anti-neutrophil cytoplasmic autoantibodies (p-ANCA) have been found in 26% to 85% of PSC patients and up to 68% of UC patients (42, 43). In recent years, an increasing number of studies have noted that rheumatoid arthritis (RA) tended to cluster with IBD (44, 45) and that patients with IBD are more likely to develop RA (46). In addition, anemia is one of the most common extraintestinal systemic complications of IBD, affecting approximately 41-75% of children with IBD (47). The etiology of IBD anemia is multifactorial, which may be mainly chronic inflammation and intestinal mucosal damage leading to insidious chronic blood loss (48), mainly involving iron deficiency anemia and chronic disease anemia. More importantly, increased the risk of colorectal cancer (CRC) in IBD patients has been attributed to long-standing chronic inflammation, with the contribution of genetic alterations and environmental factors such as the microbiota (49), but the detailed mechanism is still elusive (50). IBD-associated CRC (IBD-CRC) might appear through different tumorigenesis pathways, because tumors occur in the inflamed area of the colon and have characteristic clinicopathological features (51, 52).

There were also many words about IBD diagnostic methods in the keywords, such as endoscopy, C-reactive protein, fecal calprotectin, antibody, CT and so on. Endoscopic biopsy was the gold standard for determining the diagnosis of IBD. Since mucosal healing has become an important treatment goal, colonoscopy has played an important role in monitoring disease activity (53). Moreover, colonoscopy could better monitor and manage complications of colorectal tumors, such as stenosis (53). C-reactive protein was a biomarker used to monitor disease activity, but it had a poor correlation with endoscopy results, and one-third of patients never showed an increase in concentration (54). About 60-70% of patients’ serum might contain antimicrobial antibodies, the most common being the anti-Saccharomyces cerevisiae antibody (ASCA) IgA (43, 55). The sensitivity and specificity of these antibodies were too low for diagnostic purposes, such as ASCA (43), p-ANCA (43, 56), anti-ganglioside antibodies (57), etc., but these antibodies could be applied to the clinical diagnosis of other autoimmune diseases (58), such as ASCA in most patients with celiac disease (43, 59). The study showed that ASCA positivity had been found in 75% of CD patients and 27% of UC patients, and the p-ANCA was detected in 72% of UC patients and 16% of CD patients, so ASCA and p-ANCA could be used to identify UC and CD (43). Other biomarkers such as microRNA could also be used for the identification of UC and CD (60). Fecal biomarkers, including fecal calprotectin, were increasingly used as screening tests and assessing disease activity in IBD. Fecal calprotectin, a protein that could be detected in feces, whose concentration was related to the infiltration of neutrophils in the intestine, was a surrogate marker of intestinal inflammation, and could be used to monitor disease activity, evaluate treatment response, predict clinical recurrence and postoperatively relapse (61). Therefore, it had high sensitivity and specificity for the diagnosis of IBD, because patients with low fecal calprotectin had less than 1% chance of developing IBD (62). Imaging examinations such as ultrasonography, CT or MR enterography were becoming more and more important in the treatment of CD. CT or MR enterography should be performed at diagnosis to assess the extent of the disease and whether there were complications such as stenosis or fistula, thereby providing information about disease behavior (63). During follow-up, imaging was increasingly used to assess disease activity, complications, and response to treatment (64, 65). In general, imaging had a limited role in determining the diagnosis of UC. For patients with acute severe UC, toxic megacolon should be assessed by plain abdominal radiographs (64). CT and MRI might show a thickened cardia colon, but their sensitivity or specificity was not sufficient as a diagnostic tool (64). The above diagnosis methods had certain reference value, but the accuracy needed to be further explored and resolved. Therefore, it was of great significance to study the precise diagnosis and treatment mechanism of IBD in depth.

In addition, the analysis of 96 research literature on IBD precision diagnosis and management showed that in the era of individualization, precision medicine (PM) was the future direction. PM is one of the five focus areas proposed by the Crohn’s and Colitis Foundation as a “Challenge in IBD” that deserves dedicated research (66). The burden of IBD is increasing, and because it is a disease with high morbidity and high medical costs, it puts pressure on the medical system, which highlights the need for novel and effective treatment strategies for IBD. Therefore, PM has been identified as a key strategy to improve the clinical outcome of IBD (66, 67). PM aims to use the biological characteristics of individual patients to formulate the right treatment plan for the right patient at the right time (68, 69). This requires understanding the functions of individual biological components and their multi-factor interactions on the overall impact of patient stratification (70). It is expected that the multi-omics method may be more robust in guiding the precise treatment of IBD and other complex diseases (71, 72). With the exponential increase in the availability of molecular data utilized through omics technology, network biology could become a valuable tool for analyzing patient data from multiple omics to achieve the key goals of PM for IBD and other complex diseases (73, 74). In addition, artificial intelligence, including machine learning and deep learning, could provide many potential solutions for the treatment of IBD (75). However, the current research literature on PM for IBD is scarce, and its individualized precision diagnosis method and management mechanism need to be further explored to obtain the support of a large amount of basic research data.

This study has some limitations. First, this study only focuses on publications in the WoSCC database, excluding other databases, such as PubMed and Scopus, which might produce slightly different results. And although WoSCC is a comprehensive and popular online database in the field of scientometrics, it is possible that several papers on this topic have been published in journals not included in the Web of Science. Second, non-English publications are excluded, so landmark articles published in other languages are not taken into account, resulting in a small number of articles not being included. Finally, the number of citations cannot fully reflect the quality of the publication, because it takes time to cite the manuscript. Older journals may receive more citations, so influential manuscripts might take several years to generate citations.

## 5 Conclusions

As far as we know, this research is the first bibliometric analysis of publications in the field of IBD diagnosis using visualization software and data information mining, and has obtained the current status, hotspots, and development of this field. Research in this field mainly focuses on IBD-related diseases, and the future research hotspot might be the precision medicine of IBD, but the mechanism needs to be explored in depth to provide a theoretical basis for its clinical application. IBD researchers and practitioners can use the study results to improve their understanding of the area and can catalyze their further knowledge development. Then, it can inform novice researchers, interested readers, or research managers and evaluators without specific knowledge and help them to develop a perspective on the IBD diagnosis. Finally, the study output can serve as a guide to further research and a starting point for more formal knowledge synthesis endeavors like systematic reviews and meta-analyses.

## Data availability statement

The original contributions presented in the study are included in the article/**Supplementary Material**. Further inquiries can be directed to the corresponding author.

## Author contributions

(I) Conception and design: CL, RY, and JZ. (II) Administrative support: WD. (III) Provision of study materials or patients: CL, RY, and JZ. (IV) Collection and assembly of data: SW, FX, YG, PH, and LS. (V) Data analysis and interpretation: CL, RY, and JZ. (VI) Manuscript writing: all authors. (VII) Final approval of manuscript: all authors.

## Funding

The National Natural Science Foundation of China (No. 82170549 and 82000521) and Key Science and Technology Project of Henan Science and Technology Department (No. 212102310037) funded this manuscript.

## Acknowledgments

The authors would like to thank editors and the anonymous reviewers for their valuable comments and suggestions to improve the quality of the paper.

## Conflict of interest

The authors declare that the research was conducted in the absence of any commercial or financial relationships that could be construed as a potential conflict of interest.

## Publisher’s note

All claims expressed in this article are solely those of the authors and do not necessarily represent those of their affiliated organizations, or those of the publisher, the editors and the reviewers. Any product that may be evaluated in this article, or claim that may be made by its manufacturer, is not guaranteed or endorsed by the publisher.
