# Supplementary material for: Research hotspot and trend analysis in the diagnosis of inflammatory bowel disease: A machine learning bibliometric analysis from 2012 to 2021
Source: Front Immunol. 2022 Sep 14;13:972079. doi: 10.3389/fimmu.2022.972079 (PMC9516000; doi:10.3389/fimmu.2022.972079)
Supplement: Supplementary file 2 [file Table_2.docx]

Supplement Table 2. Top 10 ranking table of authors, journals, institutions, and countries based on WoSCC

| Rank | Related researches in nearly 10 years | | | | High reference and hotspot papers | | | | Researches on precision diagnosis and management | | | |
| --- | --- | --- | --- | --- | --- | --- | --- | --- | --- | --- | --- | --- |
|  | Author | Number | Publication | Number | Author | Number | Publication | Number | Author | Number | Publication | Number |
| 1 | Peyrin-biroulet L | 119 | Inflammatory bowel diseases | 590 | Peyrin-biroulet L | 10 | Gastroenterology | 20 | FIOCCHI C | 3 | Inflammatory bowel diseases | 11 |
| 2 | Ludvigsson JF | 113 | Journal of Crohn’s colitis | 391 | Danese S | 9 | Gut | 13 | CHEN Y | 3 | Expert review of gastroenterology hepatology | 3 |
| 3 | Shen B | 98 | World journal of gastroenterology | 331 | Dignass A | 7 | Journal of Crohn’s colitis | 12 | BEATTIE RM | 2 | Journal of Crohn’s colitis | 3 |
| 4 | Danese S | 73 | Journal of pediatric gastroenterology and nutrition | 311 | Magro F | 7 | Lancet | 11 | DANESE S | 2 | Scandinavian journal of gastroenterology | 3 |
| 5 | Colombel JF | 72 | Digestive diseases and sciences | 218 | Eliakim R | 7 | Nature reviews disease primers | 5 | DRAGONI G | 2 | World journal of gastroenterology | 3 |
| 6 | Bernstein CN | 71 | Plos one | 206 | Ng SC | 6 | Inflammatory bowel diseases | 4 | DREESEN E | 2 | American journal of gastroenterology | 2 |
| 7 | Lebwohl B | 69 | Scandinavian journal of gastroenterology | 202 | Rogler G | 6 | Clinical gastroenterology and hepatology | 3 | ENNIS S | 2 | BMC gastroenterology | 2 |
| 8 | Ye BD | 69 | Alimentary pharmacology therapeutics | 182 | Sebastian S | 6 | Journal of pediatric gastroenterology and nutrition | 3 | GISBERT JP | 2 | Clinica chimica acta | 2 |
| 9 | Li Y | 67 | Medicine | 167 | Burisch J | 5 | American journal of gastroenterology | 3 | HONIG G | 2 | Digestive and liver disease | 2 |
| 10 | Rogler G | 66 | BMC gastroenterology | 139 | Rieder F | 5 | World journal of gastroenterology | 2 | HURTADO-LORENZO A | 2 | Frontiers in microbiology | 2 |
| Rank | Related researches in nearly 10 years | | | | High reference and hotspot papers | | | | Researches on precision diagnosis and management | | | |
|  | Country | Number | Institution | Number | Country | Number | Institution | Number | Country | Number | Institution | Number |
| 1 | USA | 4178 | HARVARD UNIVERSITY | 488 | USA | 75 | HARVARD UNIVERSITY | 18 | USA | 24 | Katholieke univ leuven | 4 |
| 2 | ITALY | 1540 | MAYO CLINIC | 386 | ENGLAND | 44 | MAYO CLINIC | 16 | CHINA | 15 | Cleveland clin | 3 |
| 3 | CHINA | 1438 | UNIVERSITY OF CALIFORNIA SYSTEM | 355 | ITALY | 36 | UNIVERSITY OF CALIFORNIA SYSTEM | 16 | ITALY | 11 | Crohn’s colitis fdn | 2 |
| 4 | ENGLAND | 1264 | UNIVERSITY OF LONDON | 354 | CANADA | 34 | UNIVERSITY OF TORONTO | 15 | CANADA | 10 | Harvard univ | 2 |
| 5 | GERMANY | 842 | UDICE FRENCH RESEARCH UNIVERSITIES | 297 | GERMANY | 33 | ASSISTANCE PUBLIQUE HOPITAUX PARIS APHP | 13 | SPAIN | 10 | Hosp univ princesa | 2 |
| 6 | CANADA | 821 | CLEVELAND CLINIC FOUNDATION | 291 | FRANCE | 26 | UDICE FRENCH RESEARCH UNIVERSITIES | 13 | ENGLAND | 9 | Johns hopkins univ | 2 |
| 7 | JAPAN | 758 | KAROLINSKA INSTITUTET | 283 | NETHERLANDS | 24 | KU LEUVEN | 12 | BELGIUM | 6 | Karolinska univ hosp | 2 |
| 8 | SPAIN | 695 | UNIVERSITY OF TORONTO | 278 | BELGIUM | 19 | UNIVERSITY OF LONDON | 12 | AUSTRALIA | 5 | Monash univ | 2 |
| 9 | FRANCE | 591 | ASSISTANCE PUBLIQUE HOPITAUX PARIS APHP | 267 | DENMARK | 18 | CLEVELAND CLINIC FOUNDATION | 10 | NORWAY | 5 | Shanghai jiao tong univ | 2 |
| 10 | NETHERLANDS | 525 | INSTITUT NATIONAL DE LA SANTE | 256 | SPAIN | 18 | IMPERIAL COLLEGE LONDON | 10 | SWEDEN | 5 | Stanford univ | 2 |
