# Supplementary material for: Research hotspot and trend analysis in the diagnosis of inflammatory bowel disease: A machine learning bibliometric analysis from 2012 to 2021
Source: Front Immunol. 2022 Sep 14;13:972079. doi: 10.3389/fimmu.2022.972079 (PMC9516000; doi:10.3389/fimmu.2022.972079)
Supplement: Supplementary file 3 [file Table_3.docx]

Supplement Table 3. Top 10 ranking table of authors, cited journals, institutions, and countries based on CiteSpace

| Rank | Related researches in nearly 10 years | | | | | | High reference and hotspot papers | | | | | |
| --- | --- | --- | --- | --- | --- | --- | --- | --- | --- | --- | --- | --- |
|  | Author | Number | Country | Number | Institution | Number | Author | Number | Country | Number | Institution | Number |
| 1 | LAURENT PEYRINBIROULET | 105 | USA | 4020 | Mayo Clin | 358 | AXEL DIGNASS | 7 | USA | 75 | Mayo Clin | 13 |
| 2 | JONAS F LUDVIGSSON | 102 | ITALY | 1501 | Karolinska Inst | 246 | LAURENT PEYRINBIROULET | 5 | ENGLAND | 44 | Univ Toronto | 8 |
| 3 | BO SHEN | 95 | CHINA | 1387 | Univ Toronto | 215 | SILVIO DANESE | 5 | ITALY | 36 | Agaples Markus Hosp | 6 |
| 4 | CHARLES N BERNSTEIN | 69 | ENGLAND | 1214 | Tel Aviv Univ | 211 | F MAGRO | 4 | GERMANY | 32 | Chinese Univ Hong Kong | 6 |
| 5 | SILVIO DANESE | 64 | GERMANY | 809 | Harvard Med Sch | 179 | SHAJI SEBASTIAN | 4 | CANADA | 32 | Harvard Univ | 5 |
| 6 | BENJAMIN LEBWOHL | 62 | CANADA | 788 | Cleveland Clin | 159 | FLORIAN RIEDER | 3 | FRANCE | 26 | Univ Calgary | 5 |
| 7 | BYONG DUK YE | 61 | JAPAN | 718 | Massachusetts Gen Hosp | 154 | GERHARD ROGLER | 3 | NETHERLANDS | 24 | Broad Inst MIT & Harvard | 5 |
| 8 | GERHARD ROGLER | 60 | SPAIN | 671 | Univ Milan | 148 | DAVID C WILSON | 3 | BELGIUM | 19 | Boston Childrens Hosp | 5 |
| 9 | JEANFREDERIC COLOMBEL | 58 | FRANCE | 570 | Icahn Sch Med Mt Sinai | 146 | PAOLO GIONCHETTI | 3 | SPAIN | 18 | Hannover Med Sch | 5 |
| 10 | JAE HEE CHEON | 53 | NETHERLANDS | 506 | Univ Penn | 142 | MARCUS HARBORD | 3 | CHINA | 18 | Cleveland Clin Fdn | 4 |
| Rank | Related researches in nearly 10 years | | High reference and hotspot papers | | Researches on precision diagnosis and management | | | | | | | |
|  | Cited Journal | Number | Cited Journal | Number | Author | Number | Country | Number | Institution | Number | Cited Journal | Number |
| 1 | Gastroenterology | 6576 | Gastroenterology | 100 | CLAUDIO FIOCCHI | 3 | USA | 23 | Katholieke Univ Leuven | 4 | Gastroenterology | 76 |
| 2 | American journal of gastroenterology | 6408 | Gut | 96 | GERARD HONIG | 2 | CHINA | 15 | Cleveland Clin | 3 | Gut | 70 |
| 3 | Gut | 6124 | American journal of gastroenterology | 91 | SCI WORKSHOP STEERING COMM CA | 2 | ITALY | 10 | Athos Therapeut Inc | 2 | Inflammatory bowel diseases | 68 |
| 4 | Inflammatory bowel diseases | 5743 | New England Journal of Medicine | 90 | ANDRES HURTADOLORENZO | 2 | CANADA | 9 | Univ Groningen | 2 | American journal of gastroenterology | 59 |
| 5 | World journal of gastroenterology | 4416 | Lancet | 85 | ALAIN STINTZI | 2 | SPAIN | 8 | Univ Chicago | 2 | Alimentary pharmacology therapeutics | 56 |
| 6 | Journal of Crohn’s colitis | 4323 | Inflammatory bowel diseases | 85 | GABRIELE DRAGONI | 2 | ENGLAND | 7 | Hosp Univ Princesa | 2 | Journal of Crohn’s colitis | 49 |
| 7 | Alimentary pharmacology therapeutics | 4156 | Clinical gastroenterology and hepatology | 81 | DIMITRIOS ILIOPOULOS | 2 | BELGIUM | 6 | Karolinska Inst | 2 | Lancet | 44 |
| 8 | Lancet | 4112 | Alimentary pharmacology therapeutics | 73 | LISA A BOARDMAN | 1 | AUSTRALIA | 5 | Harvard Univ | 2 | Clinical gastroenterology and hepatology | 42 |
| 9 | New England Journal of Medicine | 4082 | Scandinavian journal of gastroenterology | 62 | JEROEN JANSEN | 1 | SWEDEN | 4 | Univ Florence | 2 | New England Journal of Medicine | 38 |
| 10 | Clinical gastroenterology and hepatology | 4001 | Journal of Crohn’s colitis | 60 | GIULIA RODA | 1 | NORWAY | 4 | Karolinska Univ Hosp | 2 | World journal of gastroenterology | 35 |
